# Helouan

**Published:** 1898-11-19

**Authors:** 


### HELOUAN.

Writing in the *Practitioner*, Dr. Page May describes the advantages of Helouan as a health resort during the winter months. He says that treatment by hot baths is essentially a summer treatment. "To rise early before breakfast and walk out in a cold November fog or driving rain, or to step from a hot bath into a winter blast or driving snow, is attended with risks that are sufficiently obvious." Hence such treatment is greatly limited by climate and by season. The diseases which are capable of relief by such forms of treatment, however, have no such limitations in regard to their occurrence, and although in some cases those who are affected by them can afford to bide their time and wait until summer and "season" come round, many cannot so wait without risking the occurrence of increased and perhaps permanent damage to the tissues. Hence the advantage in the winter of a visit to a spa like Helouan, which has its season at a time of year when most sulphur spas are shut up, or, at the best, are but obtaining a mere dole of sunshine. The virtues of sulphur spas are admitted all the world over, and those who have money and leisure at their command may just as well take them in winter at Helouan as wait for the season at Harrogate or in the Pyrenees. At the same time, while agreeing with Dr. Page May as to the advantage of taking one's sulphur in warm weather, half a loaf is better than no bread, and we cannot admit that the use of sulphur treatment in winter is quite so disastrous as is suggested in the passage we have quoted. Danger can be prevented by taking care, and in many of the "scrofulous" cases and those suffering from city life, as well as in the ordinary run of acne patients who frequent these baths, so long as active exercise can be taken, the bracing cold of winter is not an altogether evil influence.

